# Giant Fibroadenoma Growing Rapidly During Pregnancy

**DOI:** 10.5812/ircmj.9531

**Published:** 2014-08-05

**Authors:** Erdal Karagulle, Emin Turk, Ozgur Hilal Erinanc, Gokhan Moray

**Affiliations:** 1 Department of General Surgery, Faculty of Medicine, Baskent University, Ankara, Turkey; 2 Department of Pathology, Faculty of Medicine, Baskent University, Ankara, Turkey

**Keywords:** Fibroadenoma, Phyllodes Tumor, Pregnancy, Lactation

## Abstract

**Introduction:**

Giant fibroadenoma is a rare disease with unknown etiology. During pregnancy, fibroadenomas increase in size and may show lactational histologic changes. High concentrations of estrogen, progesterone, and prolactin promote the ductal growth and formation of tubuloalveolar structures. This may be a reason for the significant enlargement in this period.

**Case Presentation:**

We presented a case of giant fibroadenoma, first detected at the onset of pregnancy, which grew rapidly and was excised surgically two months after the birth. There was no marked deformity in the breast nor a need to reconstruct it, despite the giant mass was excised and the mother was lactating.

**Discussion:**

We presented a rare case of giant fibroadenoma in a lactating woman. A progressively growing mass in breast can lead to structural damages. The current management approach for giant fibroadenomas is still surgical excision.

## 1. Introduction

Fibroadenomas are typically present as firm, mobile, painless, and frequently multiple breast nodules. These tumors are common, benign breast tumors that usually affect women in second and third decades of life. Fibroadenomas are usually small and can be managed conservatively; however, 0.5-2% of these lesions will grow rapidly. Giant fibroadenomas, greater than 5 cm or 500 g, can be associated with significant deformity, raising suspicion for malignancy, and requiring surgical excision (1-3). Giant fibroadenomas can occur as unilateral macromastia without definable borders or texture differences on palpation. The incidence of primary breast cancer in women less than 20 years old is approximately 0.1%; there are reports of in situ ductal carcinoma and neoplasm in a fibroadenoma (4). These benign tumors most commonly affect women with African or Asian origins (4). The exact etiology of giant fibroadenomas is unknown, but it is probably an abnormal response to estrogen, as evidenced by their increased frequency during puberty and responsiveness to pregnancy, oral contraceptives, and cyclic hormones (3, 5). We presented a case of a rapidly growing mass in the left breast during pregnancy and lactation.

## 2. Case Presentation

**Figure 1.**
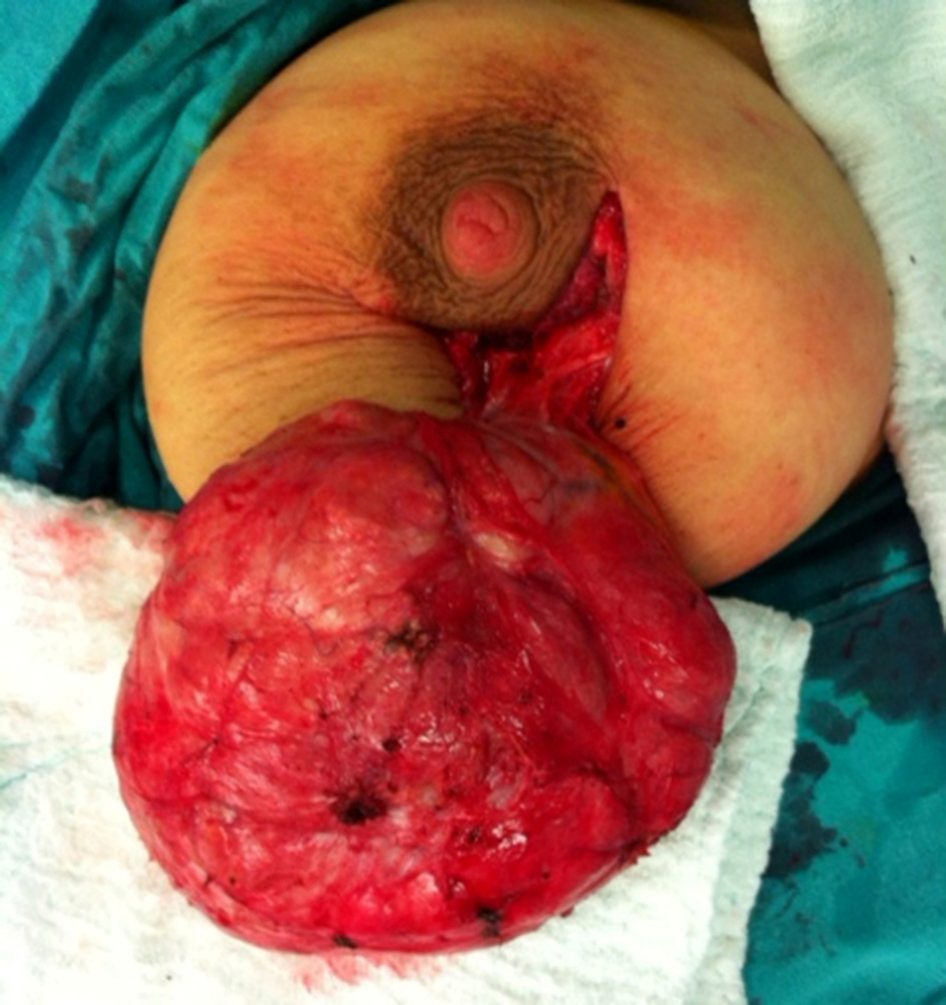
The Appearance of the Encapsulated and Lobulated Mass Excised by Surgery

**Figure 2.**
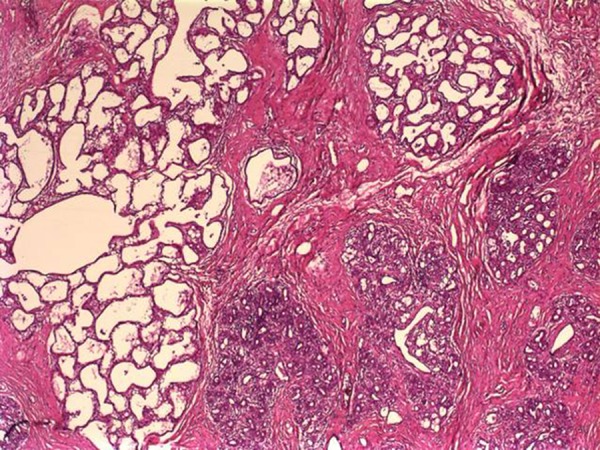
The Microscopic Section Revealing Sclerosing Adenosis in a Fibroadenoma There are large and irregularly-shaped lactation glands in the left part of the figure (H&E × 40).

A 27-year-old female patient referred with swelling in the left breast. The patient, who lived in the city of Konya, Turkey, was admitted to the hospital on 11 September 2012. Her past history was notable regarding a two-week pregnancy and giving birth to two children. Her family history was negative for breast cancer. Physical examination showed a smooth-contour, mobile, painless mass, about 4 cm, at the border of the areola at the six o’clock position in left breast. Ultrasonographic size of the mass was 42 × 22 × 13 mm. The mass was lobulated, heterogeneous, hypoechoic and solid, and was first identified as a fibroadenoma. As she was at the first trimester of her pregnancy, medical follow-up was scheduled. She did not attend her subsequent follow-ups and returned with a full-grown mass at the eighth month of pregnancy. A 15 cm, lobulated, mobile mass in the left breast was detected, extending from the areola to the inferior part of the breast. Ultrasonographic examination revealed a mass, 55 × 125 × 61 mm, which was primarily defined as a phyllodes tumor. She avoided performing excision because of pregnancy. Therefore, a tru-cut biopsy was performed. Pathological examination showed fibrosis and adenosis. The patient returned two months after giving birth, while lactating. The ultrasonographic examination result revealed a mass, 124 × 151 × 63 mm, which was heterogeneous, hypoechoic, and solid. She was again offered excision. This time she accepted excision and underwent operation under general anesthesia. The mass was completely excised (Figure 1). The mass was 580 g and 14 × 12 × 6 cm. Pathological examination revealed diffuse adenosis and complex fibroadenosis, showing lactational changes (Figure 2). After the operation it showed no prominent deformity. Milk oozing from the lateral edge of the circumareolar incision continued for two months after the operation and then stopped. Physical and ultrasonographic examinations 13 months after the operation (14 November 2013) did not show mass in the breast and the patient continued the routine follow-ups.

## 3. Discussion

Fibroadenomas are benign neoplasms with epithelial (glandular) and stromal (fibrous) components, which predominantly occur in young women (3, 4). In giant fibroadenomas, a prominent glandular epithelium and an increased stromal cellularity are described (6). Fibroadenomas are usually unilateral (90%); the masses frequently occur in the upper outer quadrant and vary in size from less than 1 cm to 20 cm (3). During pregnancy, fibroadenomas increase in size and may show lactational histologic changes. High concentrations of estrogen, progesterone, and prolactin promote ductal growth as well as formation of tubuloalveolar structures. This may be a reason for the significant enlargement in this period (7). Breast enlargement can occur in as short as a few weeks, and the mass can double in size within three to six months, growing larger than the existing normal breast tissue (8, 9). The standard treatment of all giant fibroadenomas is surgical excision (2). Local recurrence is not common, but can occur (8). On imaging, giant fibroadenomas appear as well-circumscribed masses on mammography and solid on ultrasound (10).

Some authors discuss on extirpation of these tumors before pregnancy while others tend to prefer the conservative treatment, as fibroadenomas are benign neoplasms (11). Giant fibroadenomas are typically considered as indicators of malignancy (ulceration, skin dimpling, nipple inversion, peau d’orange), compounding the need to perform tissue diagnosis in these patients (12). Tissue diagnosis in giant fibroadenomas is usually by excision rather than percutaneous biopsy, since percutaneous biopsy fails to address the mass effects or diagnose many of these cases. Other tumor entities with possible similar characteristics are phyllodes tumor, lipoma, abscess, juvenile breast hypertrophy, hamartoma, breast abscess, macrocyst, pseudoangiomatous stromal hyperplasia, or even primary breast cancer (13). Treatment modalities and the prognosis differ quite significantly in these various conditions. Benign low phyllodes tumor, the main differential diagnosis of a giant fibroadenoma, can occur at any age with a peak incidence around the fourth decade of life. They carry a higher incidence of malignancy with high-grade lesions histologically similar to sarcomas, explaining their original name of cystosarcoma phyllodes (12). It is important to note that phyllodes tumors (benign and malignant) frequently cause lymphadenopathy due to reactive changes and this should not be considered a sign of malignancy in these patients. Ultrasonography, mammography, magnetic resonance imaging or fine needle aspirations have not been helpful in definitely differentiating fibroadenoma from phyllodes tumor (4). While the phyllodes tumor should be surgically removed with safety margins, giant fibroadenomas are well encapsulated and should be enucleated. Other causes confused with giant fibroadenomas are rare and their differentiation is relatively easy.

We presented a case report of a giant fibroadenoma with an onset at early pregnancy, which grew rapidly during pregnancy and was excised surgically two months after the labor. There was no marked deformity in the breast nor a need to reconstruct it, despite the giant mass was excised and the mother was lactating. The hormonal status during pregnancy and lactation may lead to fibroadenomas growth. A progressively growing mass in breast can lead to structural damages. However, removal of a large mass from breast can cause aesthetic disorders. Surgical intervention may be operated during lactation. The current management approach for giant fibroadenomas is still surgical excision.
